# Complete Genomic Sequence of an Australian Sindbis Virus Isolated 44 Years Ago Reveals Unique Indels in the E2 and nsP3 Proteins

**DOI:** 10.1128/MRA.00246-19

**Published:** 2019-05-16

**Authors:** Paul Pickering, John G. Aaskov, Wenjun Liu

**Affiliations:** a Australian Defence Force Malaria and Infectious Disease Institute, Enoggera, Australia; b Institute of Health and Biomedical Innovation, Queensland University of Technology, Brisbane, Australia; KU Leuven

## Abstract

The complete genome sequence of a Sindbis virus (SINV) strain (SINV_AUS_1975_18953) isolated in Australia in 1975 from Culex annulirostris mosquitoes revealed unique deletions in amino acid positions 182 to 184 and 201 to 228 of the E2 envelope protein and multiple indels in the nonstructural protein 3 (nsP3).

## ANNOUNCEMENT

Sindbis virus (SINV) is a single-stranded positive-sense RNA virus of the genus *Alphavirus* in the *Togaviridae* family (1). The viral genome of the prototype strain AR339 contains 11,703 nucleotides (nt) with a 5′ cap and a 3′ poly(A) tail. The virus is maintained in nature in transmission cycles involving principally birds and *Culex* mosquitos, although it has been isolated from a variety of mosquito and vertebrate hosts (2). Humans can be infected by spillover of virus, resulting in disease with relatively benign symptoms, such as fever, rash, arthralgia, and myalgia (3–5).

SINV strain SINV_AUS_1975_18953 was isolated in suckling mice from a pool of Culex annulirostris mosquitoes collected in a forested area near Charleville (26.4°N, 146.2°E), Queensland, Australia, in 1975 (2). The mouse brain stock was used to infect cultures of C6/36 Aedes albopictus cells for 3 days at 30°C. The culture supernatant was recovered, and cell debris was removed by centrifugation at 5,000 × *g* for 10 minutes. The resulting supernatant was stored at –80°C and employed as a source of virus. Viral RNA was extracted from 140 μl of this tissue culture supernatant using the QIAamp viral RNA minikit (Qiagen, USA) and reverse transcribed using random hexanucleotide primers (Promega, USA) and SuperScript III reverse transcriptase (Invitrogen, USA), according to the manufacturer’s instructions. Overlapping fragments of 1.0 to 1.5 kb of the resultant cDNA were amplified by using Expand long template DNA polymerase (Roche) and virus-specific primers corresponding to the sequence of SINV strain MRE-16 (GenBank accession numbers AF492770 and U90536). The reverse transcription-PCR (RT-PCR) amplicons were sequenced by using dideoxy dye chain-termination Sanger sequencing technology at the Australian Genomic Research Facility. The set of Sanger sequences were read from ABI files and trimmed to remove the poor-quality reads of 5′ and 3′ ends by the quality value, and the trimmed reads were aligned to a reference SINV sequence (strain AR339, GenBank accession number J02363) to generate the whole-genome sequence with 100% coverage using the Geneious software package version 11.2.2. The nucleotide sequences in the 5′ untranslated region (UTR) and the 3′ UTR of SINV_AUS_1975_18953 were determined by Sanger sequencing of the reverse transcription and PCR amplicons using the rapid amplification of cDNA ends (RACE) strategy described previously (6).

The genome of SINV_AUS_1975_18953 contained 11,600 nt, with a G+C content of 51.8%. At the nucleotide level, the whole-genome sequence of SINV_AUS_1975_18953 shared 74.1% similarity with the prototype strain of SINV AR339 recovered from a pool of trapped mosquitoes in Sindbis Village, Egypt, in 1955 (7), 72.4% similarity with an Australian strain of SINV, SW6562 (GenBank accession number AF429428), isolated in 1984, and 95.1% similarity with an isolate of SINV MRE-16 (GenBank accession numbers AF492770 and U90536) obtained in Malaysia from Culex tritaeniorhynchus mosquitoes in 1975 (8). It also shared 90.1% similarity with a SINV virus strain isolated in China in 2013 (YN_222/China/2013, GenBank accession number MH229928).

**FIG 1:**
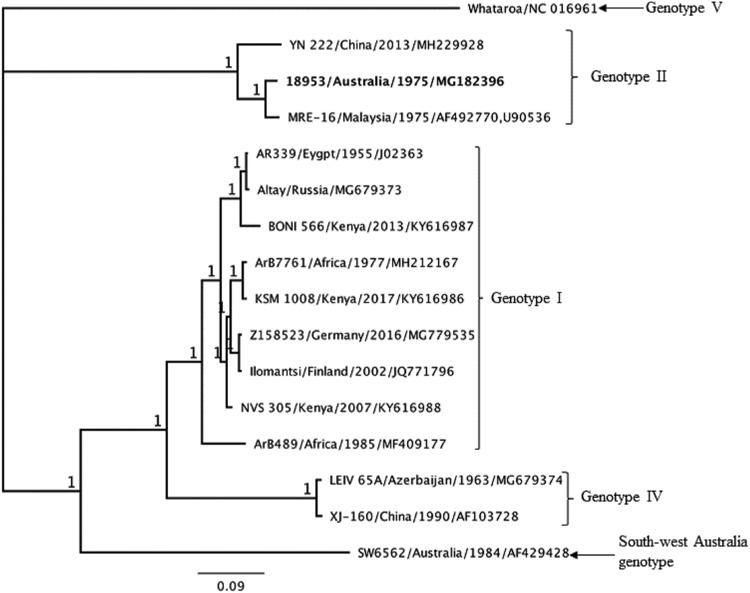
The maximum clade credibility tree of the genetic relationships between SINV_AUS_1975_18953, MRE-16/Malaysia/1975, and YN-222/China/2013 and other strains of SINV virus. The maximum clade credibility tree was generated using the Bayesian phylogenetic method in the Geneious software package version 11.1.2 for analysis of the selected whole-genome SINV sequences, employing the HKY85 substitution model, the gamma rate variation, and a chain length of 1,000,000 with a burn-in length of 10,000. Numbers at the nodes indicate posterior probabilities. The naming convention for the viruses is name of strain/country/year of isolation/GenBank accession number.

A maximum clade credibility phylogenetic analysis of the whole-genome sequences of SINV_AUS_1975_18953 and those in GenBank placed this virus in a distinct clade in SINV virus genotype II, along with SINV MRE-16/Malaysia/1975 and YN-222/China/2013 (Fig. 1).

The deduced amino acid sequences of the open reading frames of SINV_AUS_1975_18953 contained two significant deletions in the E2 protein between amino acids 182 and 184 and between 201 and 228, as well as indels in the nonstructural protein 3 (nsP3) compared to the prototype strain AR339 (Table 1). These deletions in domain B of E2 are the putative cell receptor recognition site involving virus entry (9). Similar but slightly different deletions in the E2 protein were observed in a small-plaque variant of the SINV MRE-16/Malaysia/1975 strain with a deletion between amino acids 200 and 229 and in an SINV strain of YN-222/China/2013 with a deletion between amino acids 199 and 228. The deletion of an amino acid sequence between amino acids 200 to 229 of the E2 protein in MRE-16/Malaysia/1975 virus significantly reduced its ability to infect Aedesaegypti mosquitoes by the oral route compared to its E2-undeleted parental virus (8).

**TABLE 1:** Amino acid indels in Sindbis virus strain SINV_AUS_1975_18953 compared with the prototype strain AR339 (GenBank accession no. J02363)

Gene	Amino acid positions	Amino acid sequences	
		Insertion(s)	Deletions
nsP3	329–330		PE
	353–373	VTDVSLDVEGGHVAANRSEVHSE	
	395	V	
	431	Q	
	464–465	PS	
	481–507		GGVSMSLGSIFDGETARQAAVQPLATG
	512–513		PM
	571	Q	
E2	182–184		ESS
	201–228		CKCGDYKTGTVSTRTEITGCTAIKQCVA

It is unclear how or why these deletions occurred in the E2 protein of SINV strains sampled over decades and from locations thousands of kilometers apart. It also is unclear whether SINV_AUS_1975_18953 contained only this deletion mutant, rather than it being a minor subpopulation, as was the case with the Malaysian SINV MRE-16, because the original isolation had been made in the neurological tissues of mice. The significance of the indels in the nsP3 protein also remains to be determined. Duplication of elements in this region of the nsP3 protein of another alphavirus, Ross River virus, was associated with fitness gain and an explosive outbreak of infection in the Pacific regions (10).

### Data availability.

The SINV genome sequence in this announcement is available in GenBank as accession number MG182396.
